# Prevalence and Associated Factors of Diarrhea Among Under‐Five Children in Maraki Health Center, Gondar Zone, Amhara Region, Ethiopia: A Cross‐Sectional Study

**DOI:** 10.1002/hsr2.72996

**Published:** 2026-09-01

**Authors:** Mequanente Dagnaw, Meera Indracanti

**Affiliations:** ^1^ Department of Epidemiology and Biostatistics, Institute of Public Health University of Gondar Gondar Ethiopia; ^2^ Department of Medical Biotechnology, Institute of Biotechnology University of Gondar Gondar Ethiopia; ^3^ Department of Nursing GT College Gondar Ethiopia; ^4^ Department of Medical Biotechnology, School of Allied and Healthcare Sciences Malla Reddy University Hyderabad Telangana India

**Keywords:** associated factors, childhood diarrhea, Ethiopia, prevalence, sanitation and hygiene, under‐five children, water

## Abstract

**Background and Objective:**

Diarrheal disease remains one of the leading causes of morbidity and mortality among under‐five children, particularly in low‐ and middle‐income countries. Despite ongoing efforts to improve water, sanitation, and hygiene (WASH) conditions, childhood diarrhea continues to be a major public health concern in Ethiopia. This study aimed to assess the prevalence of diarrhea and associated factors among under‐five children attending Maraki Health Center, Gondar Zone, Amhara Region, Ethiopia.

**Methods:**

An institution‐based cross‐sectional study was conducted among 361 mothers or primary caregivers of under‐five children attending Maraki Health Center in 2024. Participants were selected using systematic random sampling. Data were collected through face‐to‐face interviews using a structured interviewer‐administered questionnaire. Data were coded, checked for completeness, entered into Epi Info version 7.2.7, and exported to SPSS version 25 for cleaning and analysis. Descriptive statistics were used to summarize participant characteristics and the prevalence of diarrhea. Bivariable and multivariable logistic regression analyses were performed to identify factors associated with childhood diarrhea. Variables with a *p* value < 0.25 in the bivariable analysis were entered into the multivariable model. Multicollinearity was assessed using the Variance Inflation Factor (VIF), and model fitness was evaluated using the Hosmer–Lemeshow goodness‐of‐fit test. Statistical significance was declared at *p* < 0.05, and adjusted odds ratios (AORs) with 95% confidence intervals (CIs) were used to measure the strength of associations.

**Results:**

The prevalence of diarrhea among under‐five children during the 2 weeks preceding the survey was 28.8% (104/361; 95% CI: 24.1%–33.5%). Maternal illiteracy (AOR = 3.78, 95% CI: 1.44–9.87), maternal age of 18–25 years (AOR = 2.40, 95% CI: 1.60–3.80), rural residence (AOR = 3.90, 95% CI: 2.20–6.80), use of unimproved water sources (AOR = 3.90, 95% CI: 2.71–5.21), handwashing with water only (AOR = 1.80, 95% CI: 1.21–3.13), female sex of the child (AOR = 2.80, 95% CI: 1.45–4.12), and child age ≤ 11 months (AOR = 3.78, 95% CI: 1.44–5.87) were significantly associated with childhood diarrhea.

**Conclusion:**

The prevalence of childhood diarrhea in the study area was high. Maternal education, maternal age, place of residence, source of drinking water, handwashing practices, child sex, and child age were significant predictors of diarrhea. Strengthening maternal health education, improving access to safe drinking water, and promoting proper handwashing practices, particularly among rural households, may help reduce the burden of childhood diarrhea among under‐five children.

**Abbreviations**
AIDS: acquired immune deficiency syndrome
BF: breast feeding
EDHS: Ethiopian demographic and health survey
Epi Info: epidemiological information
HE: health education
MDG: millennium development goals
ORS: oral rehydration solution
SD: standard deviation
SPSS: statistical package for social science
SSA: Sub Saharan Africa
VIP: ventilated improved pit
WHO: World Health Organization

## 1 Introduction

Diarrheal disease remains one of the leading causes of morbidity and mortality among children under 5 years of age worldwide. According to the World Health Organization (WHO), diarrhea is defined as the passage of three or more loose or watery stools within a 24‐h period and continues to be a major public health challenge, particularly in low‐ and middle‐income countries [1]. Globally, diarrhea is responsible for approximately 443,000 deaths annually among children under 5 years of age and remains the second leading infectious cause of child mortality despite substantial progress in prevention and treatment strategies [1, 2]. Recurrent diarrheal episodes contribute not only to mortality but also to malnutrition, impaired growth, poor cognitive development, and increased susceptibility to other childhood illnesses [3].

The burden of childhood diarrhea is disproportionately concentrated in sub‐Saharan Africa and South Asia, where inadequate access to safe drinking water, poor sanitation facilities, and suboptimal hygiene practices continue to expose children to enteric pathogens [4]. Studies conducted across African countries have consistently identified unsafe water sources, poor handwashing practices, maternal illiteracy, rural residence, and younger child age as major determinants of diarrheal disease among under‐five children [5, 6]. Although substantial investments have been made in Water, Sanitation, and Hygiene (WASH) interventions, the burden of diarrhea remains high in many low‐resource settings, highlighting persistent inequalities in access to essential environmental health services.

Ethiopia continues to experience a substantial burden of childhood diarrhea despite notable improvements in child survival over the past two decades. According to the Ethiopia Demographic and Health Survey (EDHS) 2016, approximately 12% of under‐five children experienced diarrhea during the 2 weeks preceding the survey, making it one of the most common childhood illnesses in the country [7]. More recent analyses of national survey data have demonstrated that childhood diarrhea remains strongly associated with socioeconomic, environmental, and demographic factors, including maternal education, household wealth, sanitation facilities, source of drinking water, and place of residence [8, 9]. Furthermore, systematic reviews and meta‐analyses conducted in Ethiopia have reported considerable regional variations in diarrhea prevalence, indicating the continued need for locally generated evidence to guide interventions [10].

Several strategies have been implemented globally and nationally to reduce the burden of childhood diarrhea, including promotion of exclusive breastfeeding, oral rehydration therapy, rotavirus vaccination, improved sanitation, safe water supply, and handwashing with soap [11]. In Ethiopia, the Health Extension Program and the National Hygiene and Environmental Health Strategy have emphasized community‐based interventions aimed at improving sanitation coverage and hygiene behaviors. Despite these efforts, diarrhea remains among the leading causes of outpatient visits, hospital admissions, and preventable deaths among children under 5 years of age [12].

Although numerous studies have examined childhood diarrhea in Ethiopia, important knowledge gaps remain. First, many available studies are community‐based and may not reflect the burden among children seeking care in health facilities. Second, the prevalence and determinants of diarrhea vary considerably across geographical areas, socioeconomic contexts, and seasons, limiting the generalizability of findings from one setting to another [10]. Third, evidence from the Amhara Region, particularly in Gondar Zone health facilities, remains limited. Moreover, recent changes in WASH coverage, vaccination uptake, and healthcare utilization patterns necessitate updated local evidence to support targeted interventions.

What is currently known is that childhood diarrhea remains a major public health problem globally and in Ethiopia, and that factors such as unsafe drinking water, poor hygiene practices, rural residence, low maternal education, and younger child age contribute significantly to disease occurrence [5, 8, 13]. However, what remains insufficiently understood is the current magnitude of diarrhea and the relative contribution of these factors among under‐five children attending health facilities in Northwest Ethiopia. Addressing this gap is essential for designing context‐specific interventions and strengthening existing child health programs.

Therefore, this study aimed to assess the prevalence of diarrhea and its associated factors among under‐five children attending Maraki Health Center in Gondar Zone, Amhara Region, Ethiopia. The findings are expected to provide updated evidence for health professionals, policymakers, and program planners working to reduce the burden of childhood diarrhea and improve child health outcomes in the region.

## 2 Methods

### 2.1 Study Design

An institution‐based cross‐sectional study was conducted to assess the prevalence of diarrhea and its associated factors among under‐five children attending Maraki Health Center in Gondar Zone, Amhara Region, Ethiopia.

### 2.2 Study Area and Period

The study was conducted from April 19 to May 5, 2024, at Maraki Health Center, which is located in Gondar City, Central Gondar Zone, Amhara Regional State, Northwest Ethiopia. Gondar City is situated approximately 720 km northwest of Addis Ababa, the capital city of Ethiopia, and about 180 km from Bahir Dar, the capital of the Amhara Regional State. Gondar is one of the largest and oldest cities in Ethiopia and serves as an important center for education, commerce, tourism, and healthcare services in the region.

Maraki Health Center is one of the public health facilities in Gondar City and provides comprehensive primary healthcare services to the local community and surrounding areas. The health center delivers a wide range of preventive, promotive, curative, and rehabilitative services, including outpatient care, maternal and child health services, immunization programs, family planning, antenatal and postnatal care, nutrition services, and management of common childhood illnesses. The facility serves a large catchment population and receives a substantial number of under‐five children seeking healthcare services daily. The study was conducted among under‐five children and their mothers or caregivers who attended Maraki Health Center during the data collection period. The selection of the health center as the study site was based on its high patient flow and accessibility, making it an appropriate setting for assessing the prevalence of diarrhea and its associated factors among under‐five children in the study area.

### 2.3 Source and Study Population

#### 2.3.1 Source Population

All mothers or caregivers of under‐five children attending Maraki Health Center during the study period.

#### 2.3.2 Study Population

Randomly selected mothers or caregivers of under‐five children who attended Maraki Health Center and fulfilled the eligibility criteria during the data collection period.

### 2.4 Inclusion and Exclusion Criteria

#### 2.4.1 Inclusion Criteria

Mothers or caregivers of under‐five children who attended Maraki Health Center during the data collection period and were willing to participate in the study were included.

#### 2.4.2 Exclusion Criteria

Mothers or caregivers of under‐five children who were critically ill, mentally incapable of responding to the interview, or unable to communicate effectively during the data collection period were excluded from the study. Additionally, caregivers who were seriously distressed or otherwise unable to provide reliable information at the time of data collection were not included.

### 2.5 Sample Size Calculation

The sample size for this research is calculated using a single population proportion formula based on specific assumptions. This is done by taking into account a 31% prevalence rate of lifetime diarrheal disease found in a study conducted among children under five at Arba‐Minch University [14].

$$n_{0}=\frac{\left({Z_{\alpha /2})}^{2}\cdot p(1-p\right)}{d^{2}}$$

where:

$n_{0}\;$ = initial sample size

$Z_{\alpha /2}\;$ = standard normal value at 95% confidence level (1.96)

p = estimated prevalence of diarrheal disease (31%)

d = margin of error (5%)

Substituting the values:

$$n_{0}=\frac{\left({1.96)}^{2}\times 0.31(1-0.31\right)}{{\left(0.05\right)}^{2}}$$

$$n_{0}=\frac{3.8416\times 0.31\times 0.69}{0.0025}$$

$$n_{0}=\frac{3.8416\times 0.2139}{0.0025}$$

$$n_{0}=\frac{0.82171824}{0.0025}$$

$$n_{0}=328.69$$

$$n_{0}\approx 329$$

Therefore, the initial sample size (*n*_0_) = 329 participants.

If you add a 15% non‐response rate:

$$n_{f}=329+\left(329\times 0.15\right)$$

$$n_{f}=329+49.35$$

$$n_{f}=378.35$$

$$n_{f}\approx 378$$

### 2.6 Sampling Procedure

A systematic random sampling technique was employed to select study participants. First, the average number of under‐five children attending Maraki Health Center during the data collection period was obtained from the health center's registration records. The sampling interval (*K*) was then determined by dividing the estimated total number of under‐five children visiting the health center during the study period (*N* = 1131) by the required sample size (*n* = 378), using the formula *K* = 1131/378. The first participant was selected randomly from the first *K* eligible participants using a lottery method. Thereafter, every 3rd mother/caregiver of an under‐five child who attended the health center during the data collection period was included in the study until the required sample size was achieved.

When more than one eligible under‐five child was present in the same household, only one child was selected using a simple random sampling method to avoid duplication and maintain the independence of observations. Mothers or caregivers who met the inclusion criteria and provided informed consent were interviewed using the structured questionnaire.

### 2.7 Study Variables

#### 2.7.1 Dependent Variable

Diarrhea among under‐five children (yes/no), defined as the occurrence of diarrhea during the 2 weeks preceding the survey.

#### 2.7.2 Independent Variables

The independent variables included socio‐demographic, environmental and sanitation, behavioral and hygiene, child‐related and health, and diarrhea management‐related factors.

The socio‐demographic factors: it comprised the age, marital status, educational status, and occupation of the mother or caregiver, family size, monthly household income, place of residence (urban or rural), and the age and sex of the child.

Environmental and sanitation‐related factors: it included the source of drinking water, availability and type of latrine, household waste disposal methods, presence of handwashing facilities, household water treatment practices, and housing conditions.

Behavioral and hygiene‐related factors: included caregiver handwashing practices, handwashing at critical times, food handling and storage practices, child feces disposal practices, and personal hygiene practices of caregivers.

Child‐related and health factors: included breastfeeding status, history of exclusive breastfeeding, immunization status, nutritional status, recent history of illness, vitamin A supplementation, and age at initiation of complementary feeding. In addition, diarrhea management‐related factors such as health‐seeking behavior and treatment received for diarrheal illness were assessed.

### 2.8 Operational Definitions

Diarrhea: The passage of three or more loose or watery stools within a 24‐h period, as reported by the caregiver, occurring at any time during the 2 weeks preceding the survey/interview. This definition is consistent with WHO and Demographic and Health Survey (DHS) guidelines [15, 16].

Improved water source: A drinking water source that, by the nature of its design and construction, is protected from outside contamination, particularly fecal matter. Examples include piped water, public taps, boreholes, protected wells, protected springs, and rainwater collection [1].

Unimproved water source: A drinking water source not adequately protected from contamination, including unprotected wells, unprotected springs, surface water, and vendor‐provided water [1].

Improved sanitation facility: A sanitation facility that hygienically separates human excreta from human contact, including flush/pour‐flush toilets connected to sewer systems or septic tanks, ventilated improved pit latrines, pit latrines with slabs, and composting toilets [1].

### 2.9 Data Collection Tool

The data collection tool was adapted from the EDHS, World Health Organization (WHO) guidelines, and relevant literature related to childhood diarrhea. The questionnaire was designed to assess the prevalence of diarrhea and identify factors associated with diarrheal disease among under‐five children attending Maraki Health Center. The tool comprised five major sections. The first section collected information on socio‐demographic characteristics, including the age, marital status, educational level, and occupation of the mother or caregiver, family size, monthly household income, place of residence, and the age and sex of the child. The second section addressed environmental and sanitation‐related factors, such as the source of drinking water, availability and type of latrine, household waste disposal methods, presence of handwashing facilities, household water treatment practices, and housing conditions. The third section assessed behavioral and hygiene‐related factors, including caregivers' handwashing practices, handwashing at critical times, food handling and storage practices, methods of child feces disposal, and personal hygiene practices. The fourth section focused on child‐related and health factors, including breastfeeding status, history of exclusive breastfeeding, immunization status, nutritional status, recent history of illness, vitamin A supplementation, and the age at initiation of complementary feeding. The fifth section assessed diarrhea‐related information, including the occurrence of diarrhea during the 2 weeks preceding the survey, frequency and duration of diarrheal episodes, health‐seeking behavior, and treatment received for diarrheal illness. The questionnaire was initially prepared in English, translated into Amharic, and then back‐translated into English to ensure consistency and accuracy. Data were collected through face‐to‐face interviews with mothers of under‐five children, and when the mother was unavailable, the child's primary caregiver was interviewed.

#### 2.9.1 Data Collection Procedures

Data collection was conducted at Maraki Health Center using a structured interviewer‐administered questionnaire. Prior to data collection, data collectors and supervisors received training on the objectives of the study, interview techniques, ethical considerations, and the contents of the questionnaire. Eligible participants were mothers of under‐five children attending Maraki Health Center during the study period. When the mother was unavailable, the child's primary caregiver was interviewed.

Participants were selected using a systematic sampling technique until the required sample size was achieved. After obtaining informed consent, face‐to‐face interviews were conducted in a private setting to ensure confidentiality and encourage honest responses. Information was collected on socio‐demographic characteristics, environmental and sanitation factors, behavioral and hygiene practices, child‐related and health factors, and the occurrence of diarrhea among under‐five children during the 2 weeks preceding the survey.

The completed questionnaires were reviewed daily by supervisors and the principal investigator to ensure completeness, consistency, and accuracy. Any incomplete or inconsistent responses were corrected immediately through revisits or clarification from the respondents whenever possible. Throughout the data collection period, close supervision and regular monitoring were carried out to maintain the quality of the data collected.

#### 2.9.2 Data Quality Assurance

To ensure the quality of the data, several measures were implemented throughout the study. The questionnaire was adapted from the EDHS, World Health Organization (WHO) guidelines, and relevant literature on childhood diarrhea. The questionnaire was initially prepared in English, translated into Amharic, and then back‐translated into English by language experts to ensure consistency and accuracy. Prior to the actual data collection, a pretest was conducted on 5% of the total sample size at a health facility outside the study area. Based on the findings of the pretest, necessary modifications were made to improve the clarity, relevance, and consistency of the questionnaire.

Data collectors and supervisors received 1‐day training on the objectives of the study, data collection procedures, interview techniques, ethical issues, and methods of maintaining data quality. During data collection, close supervision was conducted by supervisors and the principal investigator to ensure that the questionnaires were completed correctly and consistently. Completed questionnaires were checked daily for completeness, accuracy, and consistency, and any errors or omissions were corrected promptly through consultation with the data collectors and respondents whenever possible. Data were coded and entered carefully, and regular checks were performed to identify and correct data entry errors before analysis.

#### 2.9.3 Data Management and Analysis Procedures

Data were coded, checked for completeness, and entered into Epi Info version 7.2.7 before being exported to SPSS version 25 for cleaning and statistical analysis. Data cleaning included checking for missing values, inconsistencies, and outliers. Missing data were assessed for all study variables, and no variable had more than 2.0% missing observations. Since the proportion of missing data was minimal, complete‐case analysis was performed.

Descriptive statistics, including frequencies, percentages, means, and standard deviations, were used to summarize participants' characteristics and the prevalence of diarrhea. The findings were presented using tables and charts as appropriate.

The dependent variable was the occurrence of diarrhea among under‐five children during the 2 weeks preceding the survey and was coded as 1 = yes and 0 = no. Independent variables were coded according to their respective categories, with reference groups selected based on epidemiological relevance and previous literature.

Bivariable logistic regression analysis was initially performed to assess the association between each independent variable and childhood diarrhea. Variables with a *p* value < 0.25 in the bivariable analysis were considered candidates for multivariable logistic regression. A total of 12 variables met this criterion and were entered into the multivariable model to identify factors independently associated with diarrhea while controlling for potential confounders. Adjusted odds ratios (AORs) with 95% confidence intervals (CIs) were calculated, and statistical significance was declared at *p* value < 0.05.

Prior to multivariable analysis, multicollinearity among independent variables was assessed using the Variance Inflation Factor (VIF) and tolerance statistics. Variables with VIF > 10 were considered to have significant multicollinearity. No variables were excluded due to multicollinearity. The final model showed no evidence of problematic multicollinearity, with VIF values ranging from 1.21 to 3.84.

The goodness‐of‐fit of the final logistic regression model was assessed using the Hosmer–Lemeshow goodness‐of‐fit test, which indicated adequate model fit (*χ* ^2^ = 7.42, *p* = 0.491). Model discrimination was further evaluated using the receiver operating characteristic (ROC) curve, with an area under the curve (AUC) of 0.82, indicating good discriminatory ability.

Influential observations were assessed using standardized residuals and leverage statistics. Since only one eligible under‐five child was included per participating household, within‐household correlation was not expected, and clustering adjustment was not required. Final model results were presented using adjusted odds ratios with corresponding 95% confidence intervals and *p* values. Findings were interpreted in relation to the study objectives and existing scientific evidence.

#### 2.9.4 Ethical Approval

Ethical approval was secured from the Internal Review Board (IRB) of GT College, referenced as Ref No/GC 132/05/2024, and subsequently sent to the Gondar City Health Bureau. Before initiating data collection, written informed consent was acquired from a parent and/or legal guardian, and data collection was carried out only after confirming their willingness. All ethical guidelines are detailed in the Declaration of Helsinki.

## 3 Result

### 3.1 Socio‐Economic and Demographic and Characteristics of the Households

A total of 378 mothers or caregivers of under‐five children were selected for the study. Among these, 361 participants completed the interview, yielding a response rate of 95.5%. The respondents were distributed across different age groups, with the majority being aged 20 years or older, including a considerable proportion aged 36 years and above. Most of the respondents were married. Regarding religion, almost all respondents were followers of Christianity (Table 1).

**Table 1:** Socio‐demographic and economic characteristics of mothers/caregiver of under‐five children attending Maraki health center, Gondar zone, Amhara region, Ethiopia, 2024 (*n* = 361).

Variable	Categories	Frequency	Percent
Size of the family	5 and less	168	46.5
	>5	193	53.5
No. <5 year children in the household	One child	272	75.3
	Two and above	89	24.7
Relation of the respondent to the child	Mother	343	95.00
	Care taker	18	5.00
Age of the mother or caregiver	15–24	229	63.4
	25–35	56	15.5
	36 and more	76	21.1
Marital status of the mother/caretaker	Married	322	89.20
	Divorced	22	6.10
	Single	17	4.70
Religion of parents/caretaker	Christian	186	51.50
	Muslim	115	32.00
	Protestant	60	16.50
Educational status of the mother/caretaker	Illiterate	193	53.5
	Primary	118	32.7
	Secondary and above	50	13.8
Occupation of the mother/caretaker	Farmer	69	19.10
	House wife	144	39.90
	Governmental employee	148	41.00
Age of the child's father	15–24	105	29.10
	25–35	154	42.60
	36 and above	102	28.30

### 3.2 Environmental Characteristics of the Household

In the sample of 361 participants, 289 had access to both a latrine and a hand‐washing station. The majority of households, which amounted to 249, had private latrines, and 195 of those latrines were classified as unimproved. Additionally, 217 households did not have any feces visible in the pit of their latrine. Most households dispose of their waste in a proper manner. Out of the households surveyed, 265 relied on unimproved sources of water, and many of these households spent 30 min or longer to collect water, while a significant number treated their drinking water at home (Table 2).

**Table 2:** Environmental characteristics of the households in Maraki Health Center, Amahara region, Ethiopia, 2024 (*n* = 361).

Variable	Categories	Frequency	Percent
Latrine available	Yes	289	80.1
	No	72	19.9
Type of latrine	Improved	134	37.3
	Not improved	227	62.88
Ownership of the latrine	Private	249	68.98
	Shared	112	30.02
Feces were seen in the pit hole	Yes	100	27.70
	No	261	72.30
If no latrine where they use	Open field	58	80.6
	Other	14	19.4
Hand‐washing facility	Yes	112	31.1
	No	249	68.9
Refuse disposal method	Improper	136	37.7
	Proper	225	62.3
Water source	Improved	108	29.9
	Not improved	253	70.1

### 3.3 Behavioral Characteristics of the Households

The majority of the respondents give their child other food in addition to BF. Most of the respondents prepared gruel, and of the respondents, fed their children using a cup and a spoon. Most of the respondents wash their hands using soap/ash, and water (Table 3).

**Table 3:** Behavioral characteristics of the households in Maraki health center, Amhara region, Ethiopia, 2024 (*n* = 361).

Variable	Categories	Frequency	Percent
Children take food other than breast milk	Yes	305	84.5
	No	56	15.5
Type of food	Cow's milk	120	33.24
	Gruel	132	36.57
	Adult food	109	30.19
Child feeding method	Hand	121	33.5
	Cup and spoon	240	66.5
Hand washing method	Water and soap/ash	223	61.8
	Water only	138	38.2

### 3.4 Demographic and Health Characteristics of the Indexed Children

Among the under‐five children included in the study, 178 were female, and the majority were aged 12–24 months. Regarding immunization status, 231 children had received the rotavirus vaccine, while 281 had received the measles vaccine. In this study, 60 children experienced diarrhea during the 2 weeks preceding the survey (Table 4).

**Table 4:** Socio‐demographic and health‐related characteristics of under‐five children attending Maraki health center, Gondar Zone, Amhara region, Ethiopia, 2024 (*n* = 361).

Variable	Categories	Frequency	Percent
Sex	Male	183	50.7
	Female	178	49.3
Age category	11 months or less	57	15.8
	12–23 months	108	29.9
	24–35 months	84	23.3
	35 and greater	112	31
Current breastfeeding status	Exclusive	71	19.67
	Partial	250	69.25
	No on breastfeeding	40	11.08
Age at supplementary	Less than 6 months	57	15.8
	At 6 months	232	64.3
	Greater than 6 months	72	19.9
Measles virus vaccine	Yes	281	77.8
	No	80	22.2
Rotavirus vaccine	Yes	231	63.99
	No	130	36.01
Have had diarrhea in the last 2 weeks	Yes	104	28.80
	No	216	71.20

### 3.5 Prevalence of Childhood Diarrhea

**Figure 1:**
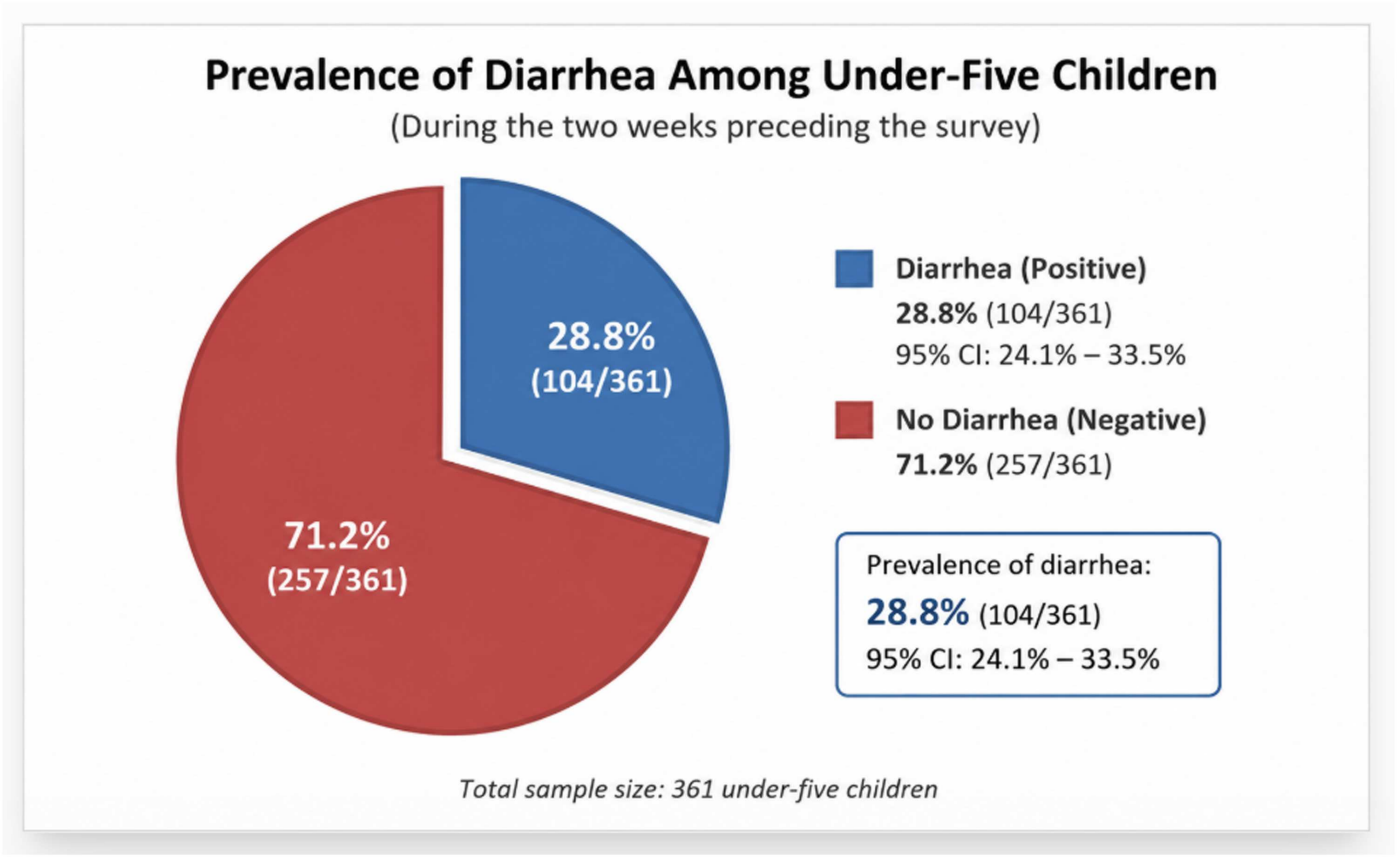
Prevalence of diarrhea among under‐five children attending Maraki Health Center, Gondar zone, Amhara region, Ethiopia, 2024 (*n* = 361).

The prevalence of diarrhea among under‐five children during the 2 weeks preceding the survey was 28.8% (104/361; 95% CI: 24.1%–33.5%) (Figure 1).

### 3.6 Factors Associated With Childhood Diarrhea

The association between socio‐demographic, environmental, behavioral, and child‐related factors and childhood diarrhea was assessed using bivariable and multivariable logistic regression analyses. Variables with a *p* value < 0.25 in the bivariable analysis were entered into the multivariable logistic regression model, and statistical significance was declared at *p* < 0.05.

The multivariable logistic regression analysis showed that maternal illiteracy (AOR = 3.78, 95% CI: 1.44–9.87), maternal age of 18–25 years (AOR = 2.40, 95% CI: 1.60–3.80), rural residence (AOR = 3.90, 95% CI: 2.20–6.80), use of unimproved water sources (AOR = 3.90, 95% CI: 2.71–5.21), handwashing with water only (AOR = 1.80, 95% CI: 1.21–3.13), female sex of the child (AOR = 2.80, 95% CI: 1.45–4.12), and child age ≤ 11 months (AOR = 3.78, 95% CI: 1.44–5.87) were significantly associated with childhood diarrhea. Children from mothers who were illiterate, younger mothers, rural households, households using unimproved water sources, and mothers who washed their hands with water only were more likely to experience diarrhea compared with their respective reference groups (Table 5).

**Table 5:** Bivariate and multivariable analysis of prevalence and associated factors of diarrhea among under‐five children in Maraki health center, Gondar Zone, Amhara region (*n* = 361).

Variables	COR (95% CI)	AOR (95% CI)	*p* value
Mother's educational status			
Illiterate	3.14 (1.27–7.77)	3.78 (1.44–9.87)	0.007
Primary	0.80 (0.43–1.64)	0.72 (0.35–1.48)	0.38
Secondary	1.79 (0.71–4.52)	2.23 (0.83–6.03)	0.11
College and above	1.00	1.00	—
Mother's age (years)			
18–25	1.9 (1.6–3.56)	2.4 (1.6–3.8)	0.02
26–35	2.1 (1.6–3.0)	1.6 (1.1–3.1)	0.03*
≥ 36	1.00	1.00	—
Residence			
Urban	1.00	1.00	—
Rural	4.0 (2.9–5.7)	3.9 (2.2–6.8)	0.01
Water source			
Improved	1.00	1.00	—
Unimproved	2.1 (1.5–2.9)	3.9 (2.71–5.21)	< 0.001
Handwashing method			
Water and soap/ash	1.00	1.00	—
Water only	1.3 (0.96–2.51)	1.8 (1.21–3.13)	0.03
Sex of child			
Female	2.3 (1.6–3.1)	2.8 (1.45–4.12)	0.01
Male	1.00	1.00	—
Age of child (months)			
≤ 11	3.14 (1.27–7.78)	3.78 (1.44–5.87)	0.04
12–23	1.79 (0.71–4.52)	2.23 (0.83–6.03)	0.06
24–35	2.37 (0.90–6.29)	2.93 (1.03–8.30)	0.04
≥ 36	1.00	1.00	—
Duration of breastfeeding			
< 6 months	1.87 (1.2–2.9)	1.1 (0.63–1.9)	0.62
6 months	1.87 (1.2–2.9)	1.1 (0.63–1.9)	0.11
> 6 months	1.00	1.00	—

* Significant at *p* value < 0.05.

## 4 Discussion

This study assessed the prevalence of diarrhea and its associated factors among under‐five children attending Maraki Health Center, Northwest Ethiopia. The prevalence of diarrhea during the 2 weeks preceding the survey was 28.8% (104/361; 95% CI: 24.1%–33.5%), indicating that diarrhea remains a major public health concern among children in the study area. Significant predictors included maternal educational status, maternal age, residence, drinking water source, handwashing practice, sex of the child, and child age.

The prevalence observed in this study is considerably higher than the global estimate reported by the World Health Organization, which indicates that approximately 9%–10% of under‐five children experience diarrhea during a 2‐week recall period in many low‐ and middle‐income settings (WHO, 2024). The finding is also higher than the prevalence reported in the EDHS, where diarrhea prevalence among under‐five children was approximately 12% [7]. However, the prevalence is comparable to findings reported from several facility‐based studies in Ethiopia, including studies conducted in Arba Minch (30.5%) [17], Debre Berhan (26.1%) [18], and another study in North West Ethiopia (27.5%) [19]. Similar prevalence estimates have been reported from other African countries, including Nigeria [20] and Kenya [21]. The relatively high prevalence observed in the current study may be explained by the facility‐based design, which tends to capture symptomatic children seeking healthcare services and therefore overestimates disease occurrence compared with community‐based surveys. Seasonal factors, variations in sanitation coverage, and differences in socioeconomic conditions may also contribute to the observed differences.

Maternal educational status was significantly associated with childhood diarrhea. Children of illiterate mothers were nearly four times more likely to experience diarrhea compared with children whose mothers had a college‐level education or above. This finding is consistent with studies conducted in Ethiopia [10, 22], Nigeria [23], Kenya [21], and Bangladesh [24]. Maternal education influences childcare practices, hygiene behavior, health‐seeking behavior, and knowledge of disease prevention. Educated mothers are more likely to practice appropriate sanitation, treat drinking water, maintain proper food hygiene, and seek timely medical care when children become ill. Consequently, educational attainment serves as an important social determinant of child health.

Maternal age was another important predictor of childhood diarrhea. Children of mothers aged 18–25 years had significantly higher odds of diarrhea than children of older mothers. Similar findings have been reported in studies conducted in Ethiopia [18] and Uganda [25]. Younger mothers may have limited experience in child feeding, hygiene maintenance, and illness recognition compared with older mothers. Older mothers often acquire practical childcare skills through experience and repeated exposure to health information, enabling them to better prevent diarrheal illnesses.

The study also found a strong association between rural residence and childhood diarrhea. Children from rural households were approximately four times more likely to experience diarrhea than those from urban households. This finding is consistent with reports from EDHS analyses [7], studies from Ethiopia [10], and other African countries [20, 26]. Rural communities frequently experience limited access to improved water sources, sanitation facilities, waste disposal services, and healthcare infrastructure. These environmental disadvantages increase children's exposure to fecal contamination and infectious pathogens, thereby elevating the risk of diarrheal disease.

Access to safe drinking water remains one of the most important determinants of childhood diarrhea. In the current study, children living in households using unimproved water sources had nearly four times higher odds of diarrhea compared with those using improved water sources. This finding agrees with evidence from WHO and UNICEF reports [11], systematic reviews conducted in Africa [4], and studies from Ethiopia [17]. Unimproved water sources are often contaminated with human and animal fecal matter, facilitating the transmission of bacterial, viral, and parasitic pathogens responsible for diarrheal diseases. The finding reinforces the importance of expanding access to safe water supplies and promoting household water treatment practices.

Handwashing practice was also significantly associated with diarrhea occurrence. Children whose caregivers washed their hands using water only were nearly twice as likely to develop diarrhea compared with those whose caregivers used soap or ash. Similar findings have been reported in studies from Ethiopia [17], Kenya [27], and Tanzania [28]. Handwashing with soap is one of the most cost‐effective interventions for preventing diarrheal diseases because it interrupts fecal‐oral transmission pathways. Caregivers who fail to use soap may inadvertently contaminate food, water, and household surfaces during child feeding and caregiving activities.

An unexpected finding of this study was the higher likelihood of diarrhea among female children. Female children had approximately three times greater odds of developing diarrhea than male children. This finding differs from many previous studies conducted in Ethiopia and other countries, where sex was either not significantly associated with diarrhea or male children experienced slightly higher prevalence [10, 22]. Therefore, this result should be interpreted cautiously. The observed association may reflect residual confounding, differences in feeding practices, healthcare utilization, age distribution, or other unmeasured sociocultural factors rather than a true biological susceptibility. Further studies are needed to explore this relationship.

Child age was another significant determinant of diarrhea. Children aged 11 months or younger were more likely to experience diarrhea than older children. Similar findings have been reported in studies from Ethiopia [17], Nigeria [20], and Bangladesh [24]. Younger children have immature immune systems and are more vulnerable to infections. In addition, the transition from exclusive breastfeeding to complementary feeding exposes infants to contaminated food, water, and feeding utensils, increasing the risk of diarrheal diseases.

## 5 Conclusions

This study found that the prevalence of diarrhea among under‐five children attending Maraki Health Center was 28.8% (104/361; 95% CI: 24.1%–33.5%), indicating that childhood diarrhea remains an important public health concern in the study area. Maternal educational status, maternal age, place of residence, source of drinking water, handwashing practices, sex of the child, and child age were significantly associated with childhood diarrhea. Specifically, children of illiterate mothers, younger mothers, rural residents, households using unimproved water sources, and caregivers who practiced handwashing with water only were at increased risk of diarrhea. Female children and children aged 11 months or younger were also more likely to experience diarrhea.

Although sanitation‐related variables, including latrine availability, type of latrine, handwashing facilities, and waste disposal methods, were not significantly associated with diarrhea in this study, these findings should be interpreted cautiously. The lack of significant associations may reflect measurement limitations, limited variability in sanitation conditions, potential misclassification, or other methodological constraints rather than the absence of a true effect. Overall, the findings highlight the need for interventions that improve maternal education, access to safe drinking water, and effective handwashing practices, particularly among rural households, to reduce the burden of childhood diarrhea.

## 6 Limitations of the Study

The findings of this study should be interpreted in light of several limitations. First, the facility‐based cross‐sectional design limits the ability to establish causal relationships and may not fully represent the broader community, as children attending healthcare facilities are more likely to be symptomatic and have different care‐seeking behaviors than children in the general population. Second, data were collected over a short period (17 days) within a single season, which may not adequately capture seasonal variations in diarrheal disease occurrence. Since the study was conducted during a specific season, the prevalence estimate may differ from that observed at other times of the year, thereby limiting the generalizability of the findings.

Third, diarrhea status was assessed based on caregivers' reports of episodes occurring during the 2 weeks preceding the survey, which may be subject to recall bias and could have resulted in under‐ or over‐reporting of diarrhea episodes. In addition, variations in respondents' understanding of the definition of diarrhea may have introduced misclassification. Fourth, several potentially important confounding factors, including household wealth status, detailed child feeding practices, and healthcare‐seeking behaviors, were not fully measured and may have influenced the observed associations. The unexpected higher odds of diarrhea among female children, for example, may reflect residual confounding from unmeasured social, behavioral, or caregiving factors rather than a true biological susceptibility. Finally, because diarrhea prevalence was relatively common in this study, the odds ratios estimated using logistic regression may have overestimated the magnitude of associations compared with prevalence ratios.

Despite these limitations, the study provides valuable evidence on the burden and determinants of childhood diarrhea in the study area and highlights important targets for intervention, including maternal education, access to safe drinking water, and improved hygiene practices.

## Author Contributions

**Mequanente Dagnaw:** conceptualization, investigation, writing – original draft, writing – review and editing, software, and data curation. **Meera Indracanti:** writing – original draft, validation, data curation.

## Funding

The authors have nothing to report.

## Ethics Statement

Ethical clearance was obtained from the Internal Review Board (IRB) of GT Technology College with the reference no Ref No/GTTC 132/05/2024, and then submitted to the Gondar City Health Bureau. All ethical principles are outlined in the Declaration of Helsinki.

## Consent

Before collecting data, informed written consent was obtained from a parent and/or legal guardian, and data collection was conducted after ensuring their willingness.

## Conflicts of Interest

The authors declare no conflicts of interest.

## Transparency Statement

The lead author, Mequanente Dagnaw, affirms that this manuscript is an honest, accurate, and transparent account of the study being reported; that no important aspects of the study have been omitted; and that any discrepancies from the study as planned (and, if relevant, registered) have been explained.

## Data Availability

The corresponding author will have the right access to the data upon request.
